# Circ_PIP5K1A regulates cisplatin resistance and malignant progression in non-small cell lung cancer cells and xenograft murine model via depending on miR-493-5p/ROCK1 axis

**DOI:** 10.1186/s12931-021-01840-7

**Published:** 2021-09-18

**Authors:** Nan Feng, Zhi Guo, Xiaokang Wu, Ying Tian, Yue Li, Yan Geng, Yan Yu

**Affiliations:** 1 Department of Clinical Laboratory, The Second Affiliated Hospital of Xi’an Jiaotong University, No.157 West Fifth Road, Xincheng District, Xi’an, 710004 Shaanxi Province China; 2 The Public Health, Xi’an Jiaotong University Health Science Center, No.76 Yanta West Road, Xi’an, 710061 Shaanxi Province China

**Keywords:** Circ_PIP5K1A, miR-493-5p, ROCK1, Cisplatin resistance, Non-small cell lung cancer

## Abstract

**Background:**

Chemoresistance limits the therapeutic effect of cisplatin (DDP) on non-small cell lung cancer (NSCLC). Circular RNAs (circRNAs) function as important regulators in chemoresistance. This study aimed to explore the regulation of circRNA Phosphatidylinositol-4-Phosphate 5-Kinase Type 1 Alpha (circ_PIP5K1A) in DDP resistance.

**Methods:**

The expression analysis of circ_PIP5K1A, micoRNA-493-5p (miR-493-5p) and Rho Associated Coiled-Coil Containing Protein Kinase 1 (ROCK1) was conducted through reverse transcription-quantitative polymerase chain reaction (RT-qPCR). Cell sensitivity was determined using 3-(4,5-dimethylthiazol-2-y1)-2,5-diphenyl tetrazolium bromide (MTT) assay. Cell proliferation and cell viability were evaluated by colony formation assay and MTT assay, respectively. Cell cycle and apoptosis detection was performed via flow cytometry. Cell motility was examined by transwell migration or invasion assay. Dual-luciferase reporter assay was applied to confirm the target binding. ROCK1 protein level was assayed via Western blot. In vivo assay was carried out using xenograft model in mice.

**Results:**

Circ_PIP5K1A level was abnormally increased in DDP-resistant NSCLC tissues and cells. Silencing circ_PIP5K1A reduced DDP resistance, proliferation, cell cycle progression and cell motility in DDP-resistant NSCLC cells. Circ_PIP5K1A directly interacted with miR-493-5p in NSCLC cells. The function of circ_PIP5K1A was dependent on the negative regulation of miR-493-5p. MiR-493-5p directly targeted ROCK1 and circ_PIP5K1A regulated the ROCK1 level via acting as a sponge of miR-493-5p. Overexpression of miR-493-5p inhibited chemoresistance and cancer progression by downregulating ROCK1 expression in DDP-resistant NSCLC cells. Circ_PIP5K1A regulated DDP sensitivity in vivo via the miR-493-5p/ROCK1 axis.

**Conclusion:**

These findings suggested that circ_PIP5K1A upregulated the ROCK1 expression to promote DDP resistance and cancer progression in NSCLC by sponging miR-493-5p.

## Background

Non-small cell lung cancer (NSCLC) is a familiar fatal malignancy that accounts for more than 80% cases of lung cancer [1]. Cisplatin (DDP) is an effective chemotherapeutic drug for various kinds of cancer, but drug resistance usually leads to treatment failure [2]. DDP-based chemotherapy has also been a first-line strategy for metastatic NSCLC [3]. Reducing DDP resistance is essential for the better treatment of NSCLC patients.

Circular RNAs (circRNAs) play important roles in cancer biology by functioning as molecular sponges of microRNAs (miRNAs) and inducing expression changes of downstream genes [4, 5]. CircRNA Phosphatidylinositol-4-Phosphate 5-Kinase Type 1 Alpha (circ_PIP5K1A, hsa_circ_0014130) contributed to the malignant progression of colon cancer via sponging miR-1273a [6] and promoted the developing process of gastric cancer by the miR-376c-3p/zinc finger protein 146 (ZNF146) network [7]. NSCLC research indicated that circ_PIP5K1A facilitated carcinogenesis and development by regulating different miRNA/mRNA axes, including miR-600/HIF-1α, miR-142-5p/insulin-like growth factor-1 (IGF-1) and miR-136-5p/B-cell lymphoma-2 (Bcl-2) [8–10]. The potential effect of circ_PIP5K1A on DDP resistance in NSCLC is still unclear.

MicroRNA-493-5p (miR-493-5p) was a tumor repressor in NSCLC progression by targeting integrin beta-1 (ITGB1) or DEAD-box helicase 5 (DDX5) [11, 12]. Gu et al. found that miR-493 decreased the DDP resistance in lung cancer by downregulating tongue cancer resistance‑related protein1 (TCRP1) [13]. Rho Associated Coiled-Coil Containing Protein Kinase 1 (ROCK1) exerted the oncogenic function in NSCLC, and it acted as the downstream target of different miRNAs (such as miR-135a, miR-335-5p, and miR-148b) [14–16]. ROCK1 was also associated with DDP resistance in lung cancer [17]. The involvement of miR-493-5p/ROCK1 axis in DDP resistance regulation has never been announced in NSCLC.

In this study, circ_PIP5K1A was hypothesized as a miR-493-5p sponge to result in the expression change of ROCK1 in NSCLC. The aim of this research was to investigate the circ_PIP5K1A/miR-493-5p/ROCK1 axis in chemoresistance and carcinogenesis of NSCLC.

## Materials and methods

### Tumor tissues

This research was authorized by the Ethics Committee of The Second Affiliated Hospital of Xi’an Jiaotong University. Tumor tissues from NSCLC patients were acquired at The Second Affiliated Hospital of Xi’an Jiaotong University. Patients without recurrence during primary DDP therapy and with recurrence beyond 6 months after chemotherapy were defined as Tumor-sensitive (n = 33). Patients with tumor progression during primary DDP therapy and recurrence within 6 months were defined as Tumor-resistant (n = 23). The physiopathologic diagnoses were affirmed by two experienced pathologists. 56 patients have afforded the informed consent for this study. Tissue samples were all preserved at − 70 °C for later use.

### Cell culture and transfection

The parental NSCLC cell lines (A549, H460) and DDP-resistant cell lines (A549/DDP, H460/DDP) were purchased from BioVector NTCC Inc. (Beijing, China). Roswell Park Memorial Institute-1640 (RPMI-1640; Sigma, St. Louis, MO, USA) was supplemented with 10% fetal bovine serum (FBS; Beyotime, Shanghai, China) and 1% antibiotics (100 × Penicillin–Streptomycin Solution, Beyotime) for cell incubation. Cell growth was in 37 °C incubator with humid air and 5% CO_2_.

1 × 10^4^ A549/DDP and H460/DDP cells were cultured in the 96-well plates overnight, and transient transfection was performed through Lipofectamine™ 3000 Kit (Invitrogen, Carlsbad, CA, USA). Small interfering RNA (siRNA) of circ_PIP5K1A (si-circ_PIP5K1A), mimic or inhibitor of miR-493-5p (miR-493-5p, anti-miR-493-5p), and the negative controls (si-NC, miR-NC, anti-miR-NC) were provided by RIBOBIO (Guangzhou, China). ROCK1 overexpression was achieved by cloning the ROCK1 sequence into the pcDNA vector (Invitrogen), and the recombinant pcDNA-ROCK1 vector was named as ROCK1. The transfection concentrations were 40 nM siRNA, 40 nM mimic, 20 nM inhibitor or 2 μg plasmid.

### Reverse transcription-quantitative polymerase chain reaction (RT-qPCR) assay

RNA isolation was carried out by TRIzol™ Reagent (Invitrogen). SuperScript™ IV First-Strand Synthesis System and SYBR™ Green One-Step qPCR Kit (Invitrogen) were used to determine the levels of circ_PIP5K1A and ROCK1. The miR-493-5p expression was quantified through TaqMan Advanced miRNA cDNA Synthesis Kit and TaqMan™ Advanced miRNA Assay (Applied Biosystems, Foster City, CA, USA). The calculation of relative expression was performed through 2^−∆∆Ct^ method [18]. Additionally, circ_PIP5K1A stability was analyzed via RT-qPCR following treatment of RNase R (GENESEED) and Actinomycin D (Sigma). The primer sequences were shown in Table 1. Glyceraldehyde-phosphate dehydrogenase (GAPDH) and U6 served as the housekeeping genes for circ_PIP5K1A/ROCK1 and miR-493-5p, respectively.

**Table 1:** Primer sequences for RT-qPCR

Name	Primer sequences
Circ_PIP5K1A	Forward: 5′-CAGGCTTCTACGCTGAACG-3′
	Reverse: 5′-ACCTGCCTGCACACAGTACA-3′
PIP5K1A	Forward: 5′-ACTTACCAGCCATCGGTCTCTG-3′
	Reverse: 5′-ACATCAGGACGACCAAGGTGAAC-3′
miR-493-5p	Forward: 5′-GCCGAGTTGTACATGGTAGG-3′
	Reverse: 5′-CAGTGCAGGGTCCGAGGTAT-3′
ROCK1	Forward: 5′-GAAACAGTGTTCCATGCTAGACG-3′
	Reverse: 5′-GCCGCTTATTTGATTCCTGCTCC-3′
GAPDH	Forward: 5′-CCACATCGCTCAGACACCAT-3′
	Reverse: 5′-TGACAAGCTTCCCGTTCTCA-3′
U6	Forward: 5′-CTCGCTTCGGCAGCACA-3′
	Reverse: 5′-AACGCTTCACGAATTTGCGT-3′

### 3-(4,5-dimethylthiazol-2-y1)-2,-diphenyl tetrazolium bromide (MTT) assay

MTT assay was adopted to examine cell sensitivity to DDP. Cells were treated with DDP of various concentrations (0 μM, 5 μM, 10 μM, 15 μM, 20 μM, 25 μM, 30 μM), and incubated with 10 μL MTT solution (Beyotime) for 3 h. Then each well was added with 10 μL formazan solving reagent (Beyotime) and the absorbance (570 nm) was measured through the microplate reader (Bio-Rad, Hercules, CA, USA). DDP concentration at 50% cell viability was termed as the maximum half inhibitory concentration (IC_50_). In addition, cell viability curves of A549/DDP and H460/DDP cells were plotted after transfection for different times (0 day, 1 day, 2 days, 3 days).

### Colony formation assay

Cell proliferation was assessed using colony formation assay. Transfection with different oligonucleotides and vectors was conducted for 24 h. Subsequently, cells were collected and transplanted into 12-well plates for two weeks. 4% paraformaldehyde and 0.1% crystal violet (Sigma) were incubated to cell colonies for 15 min, then Image J software (NIH, Bethesda, MD, USA) was applied for number counting.

### Flow cytometry

1 × 10^5^ DDP-resistant cells were harvested by trypsin (Beyotime). Cell cycle analysis and apoptosis detection were respectively performed using Cell Cycle Analysis Kit (Beyotime) and Annexin V-FITC Apoptosis Detection Kit (Beyotime), according to the producer’s instruction books. Cell determination was conducted through a flow cytometer (BD Biosciences, San Diego, CA, USA), followed by the analysis of cell distribution at different phases and the calculation of apoptotic rate.

### Transwell assay

Cell motility was determined through transwell chamber (Corning Inc., Corning, NY, USA). 5 × 10^4^ cells were pipetted into the top chamber for migration assay, and equal number of cells were seeded into the top chamber enveloped with matrigel (Corning Inc.) for invasion assay. The chamber was incubated at 37 °C for 24 h following the addition of cell medium into the bottom chamber. Then cells passed across to the membranes were measured on the inverted microscope (Olympus, Tokyo, Japan). Cell images were obtained under ×100 magnification, and the migrated or invaded cells were counted under three view of fields.

### Dual-luciferase reporter assay

The binding sites between targets were predicted via the online starbase (http://starbase.sysu.edu.cn). Circ_PIP5K1A sequence was inserted into the pmirGLO vector (Promega, Madison, WI, USA) to construct the wild-type plasmid (circ_PIP5K1A-WT). The miR-493-5p binding sites in circ_PIP5K1A sequence were mutated and the mutant control (circ_PIP5K1A-MUT) was obtained. Also, the luciferase plasmids for ROCK1 were defined as ROCK1-WT and ROCK1-MUT. DDP-resistant cells were transfected with miR-493-5p or miR-NC and each luciferase plasmid. After cell incubation at 37 °C for 48 h, luciferase activity analysis was carried out through Dual-luciferase Reporter Assay Kit (Promega).

### Western blot

Radioimmunoprecipitation assay (RIPA) containing protease inhibitor was applied for extraction of total protein. 50 μg proteins of each sample were electrophoresed on 12% TruPAGE™ Precast Gels using Sigma-Aldrich® Dual Run and Blot System (Sigma) and transferred to Immobilon-E Polyvinylidene Fluoride Membrane (Sigma) through the Trans-Blot Turbo Transfer System (Bio-Rad). The non-specific proteins were blocked and the membranes were incubated with primary antibodies of ROCK1 (Abcam, Cambridge, UK; ab97592, 1:1000) or GAPDH (Abcam, ab128915, 1:1000) at 4 °C overnight. After the incubation of Goat Anti-Rabbit IgG H&L secondary antibody (ab205718, 1:3000), immunoreactive blots were visualized via Enhanced Chemiluminescence (ECL) Substrate (Bio-Rad). GAPDH were used as the internal reference, and the protein intensity was analyzed by ImageJ software (NIH).

### Xenograft model in mice

BALB/c male nude mice were bought from Vital River Laboratory Animal Technology Co., Ltd. (Beijing, China). Lentiviral vectors (RIBOBIO) was used for stable transfection, including lentivirus negative control (lenti-NC), lentiviral circ_PIP5K1A (lenti-circ_PIP5K1A), and lentiviral short hairpin RNA of circ_PIP5K1A (sh-circ_PIP5K1A). A549/DDP cells were respectively transfected with these lentiviral vectors, followed by cell injection (2 × 10^6^ cells) into the mice with 6 mice/group. 10 days later, mice were treated with intraperitoneal injection of 6 mg/kg DDP once two days and tumor volume (length × width^2^ × 0.5) was determined every 5 days. Mice were sacrificed through the flow rate of CO_2_ after cell injection for 30 days, then tumors were dissected from mice and weighed on the electronic scale. Circ_PIP5K1A, miR-493-5p and ROCK1 levels were examined using RT-qPCR and Western blot assays. Ki67 (Sigma, SAB5600249) and Cleaved-caspase 3 (Sigma, SAB1305630) protein detection was performed by Immunohistochemistry (IHC) assay. All programs of animals were ratified by Animal Ethical Committee of The Second Affiliated Hospital of Xi’an Jiaotong University and in consistent with the Management and Use Guidelines of Laboratory Animals of NIH.

### Statistical analysis

Data were displayed as mean ± standard deviation (SD) and statistical analysis was carried out through SPSS 22.0 (SPSS Inc., Chicago, IL, USA). The relationships between gene levels in tumor samples were determined through Pearson’s correlation coefficient. The group difference was compared using Student’s *t*-test and one-way analysis of variance (ANOVA) followed by Tukey’s test. Statistically, *P* < 0.05 was termed as a significant difference.

## Results

### Circ_PIP5K1A was upregulated in DDP-resistant NSCLC tissues and cells

**Fig. 1:**
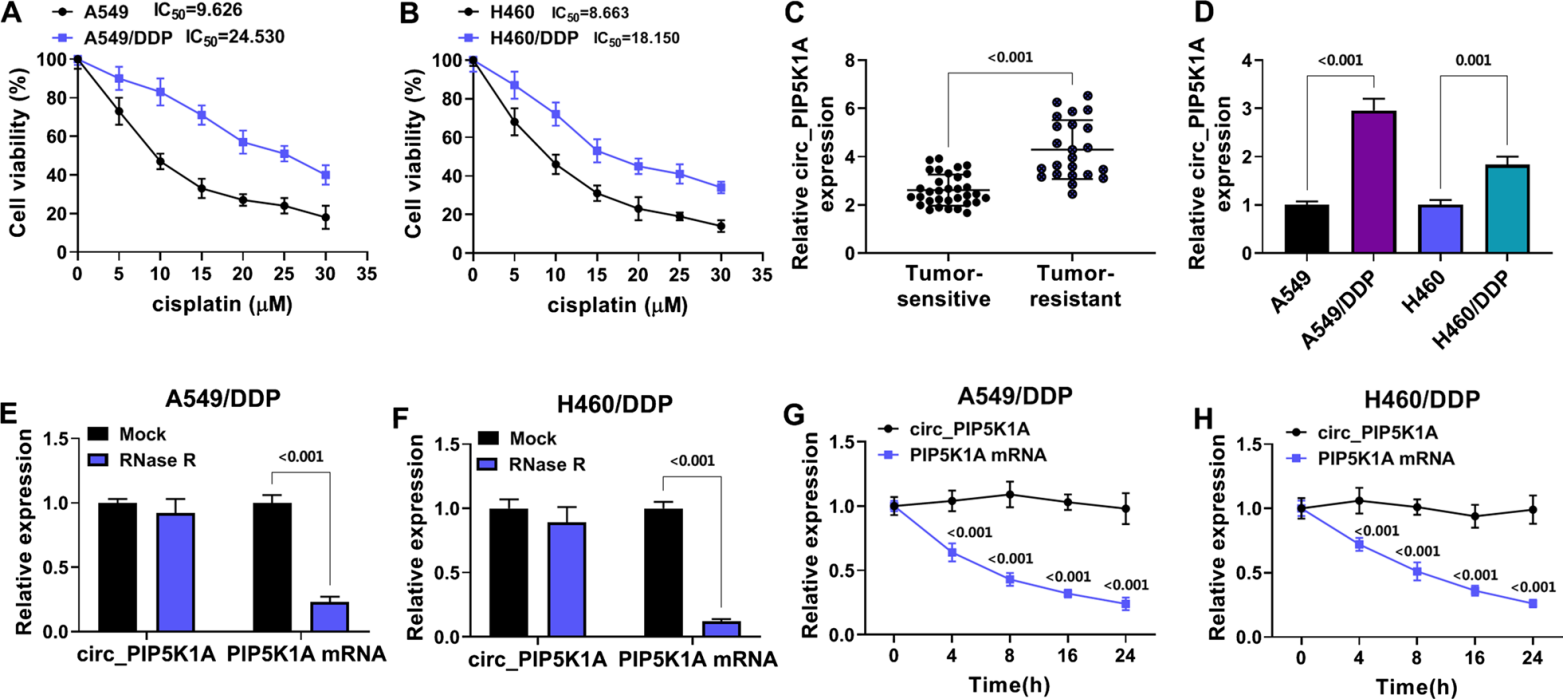
Circ_PIP5K1A was upregulated in DDP-resistant NSCLC tissues and cells. **A** IC_50_ of DDP was examined by MTT assay in A549, A549/DDP (**A**) and H460, H460/DDP (**B**) cells. **C**, **D** the circ_PIP5K1A expression was assayed by RT-qPCR in tumor-resistant tissues (**C**) and DDP-resistant cells (**D**). **E**–**H** total RNA was treated with RNase R (**E**, **F**) and cells were incubated with Actinomycin D (**G**, **H**), then circ_PIP5K1A and PIP5K1A quantification was conducted by RT-qPCR. **P < 0.01, ***P < 0.001

Cell viability analysis showed that IC_50_ value was higher in A549/DDP (IC_50_ = 24.530) and H460/DDP (IC_50_ = 18.150) cells than that in A549 (IC_50_ = 9.626) and H460 (IC_50_ = 8.663) cells, indicating that DDP resistance was presented in A549/DDP and H460/DDP cells (Fig. 1A, B). The results of RT-qPCR assay demonstrated that circ_PIP5K1A expression was obviously increased in tumor-resistant tissues relative to tumor-sensitive tissues (*P* < 0.001) and A549/DDP or H460/DDP cells compared with the A549 or H460 cells (*P* < 0.001, *P* = 0.001) (Fig. 1C, D). RNase R treatment significantly decreased the PIP5K1A mRNA level compared to the Mock group (*P* < 0.001), while the difference of circ_PIP5K1A expression between Mock and RNase R groups was not conspicuous (Fig. 1E, F). Also, the half-life of circ_PIP5K1A was much longer than PIP5K1A (*P* < 0.001) after cells were incubated to Actinomycin D (Fig. 1G, H). Circ_PIP5K1A was an upregulated circRNA in DDP-resistant NSCLC samples and cells.

### Circ_PIP5K1A knockdown inhibited DDP resistance and malignant behaviors in DDP-resistant NSCLC cells

**Fig. 2:**
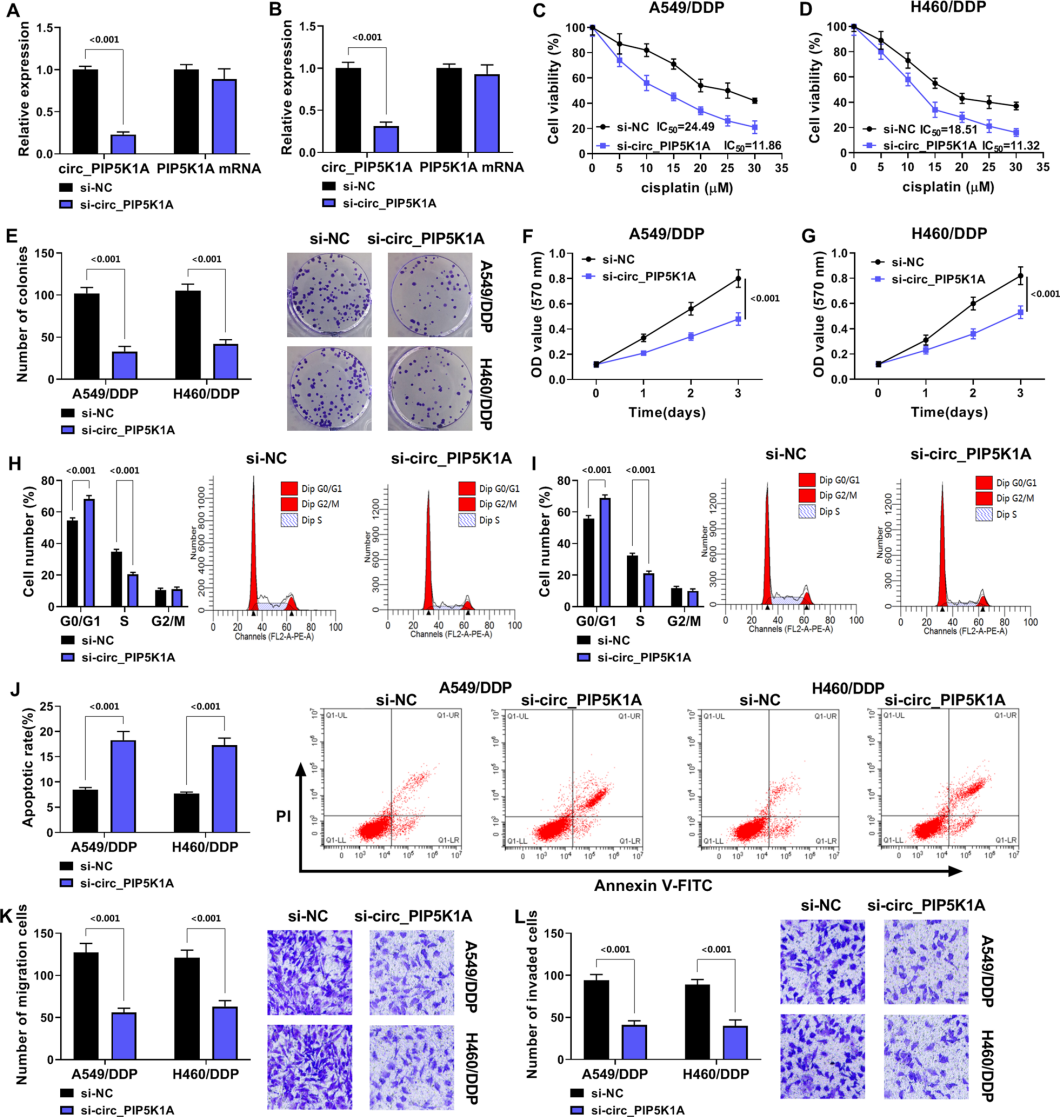
Circ_PIP5K1A knockdown inhibited DDP resistance and malignant behaviors in DDP-resistant NSCLC cells. Transfection of si-circ_PIP5K1A or si-NC was performed in A549/DDP and H460/DDP cells. **A**, **B** RT-qPCR was used to detect the levels of circ_PIP5K1A and PIP5K1A. **C**, **D** MTT assay was used to determine the IC_50_ value of DDP. **E**–**G** colony formation assay (**E**) and MTT (**F**, **G**) were used to assess cell proliferation capacity and cell viability. **H**–**J** flow cytometry was applied to examine cell cycle progression (**H**, **I**) and apoptosis (**J**). **K**, **L** transwell assay was applied to measure cell migration (**K**) and invasion (**L**). ***P < 0.001

The specific siRNA was used to inhibit the circ_PIP5K1A level. As shown in Fig. 2A, B, circ_PIP5K1A expression (*P* < 0.001) rather than PIP5K1A mRNA level was downregulated in si-circ_PIP5K1A-transfected A549/DDP and H460/DDP cells contrasted to si-NC-transfected cells. Transfection of si-circ_PIP5K1A induced an inhibitory influence on the IC_50_ value of DDP (IC_50_ = 11.86, IC_50_ = 11.32) relative to transfection of si-NC (IC_50_ = 24.49, IC_50_ = 18.51) in A549/DDP and H460/DDP cells (Fig. 2C, D). By performing colony formation assay (Fig. 2E) and MTT assay (Fig. 2F, G), we found that cell proliferation ability and cell viability were suppressed in si-circ_PIP5K1A group compared with si-NC group (*P* < 0.001). Flow cytometry revealed that si-circ_PIP5K1A resulted in cell cycle arrest from G0/G1 to S phase (Fig. 2H, I) but elevated cell apoptotic rate (Fig. 2J), by contrast with si-NC group (*P* < 0.001). The migrated and invaded cells were reduced by si-circ_PIP5K1A transfection compared with si-NC transfection (*P* < 0.001), suggesting that circ_PIP5K1A expression repression restrained cell motility in A549/DDP and H460/DDP cells (Fig. 2K, L). Thus, inhibition of circ_PIP5K1A repressed the chemoresistance to DDP and the malignant phenotypes in DDP-resistant cells.

### Circ_PIP5K1A interacted with miR-493-5p

**Fig. 3:**
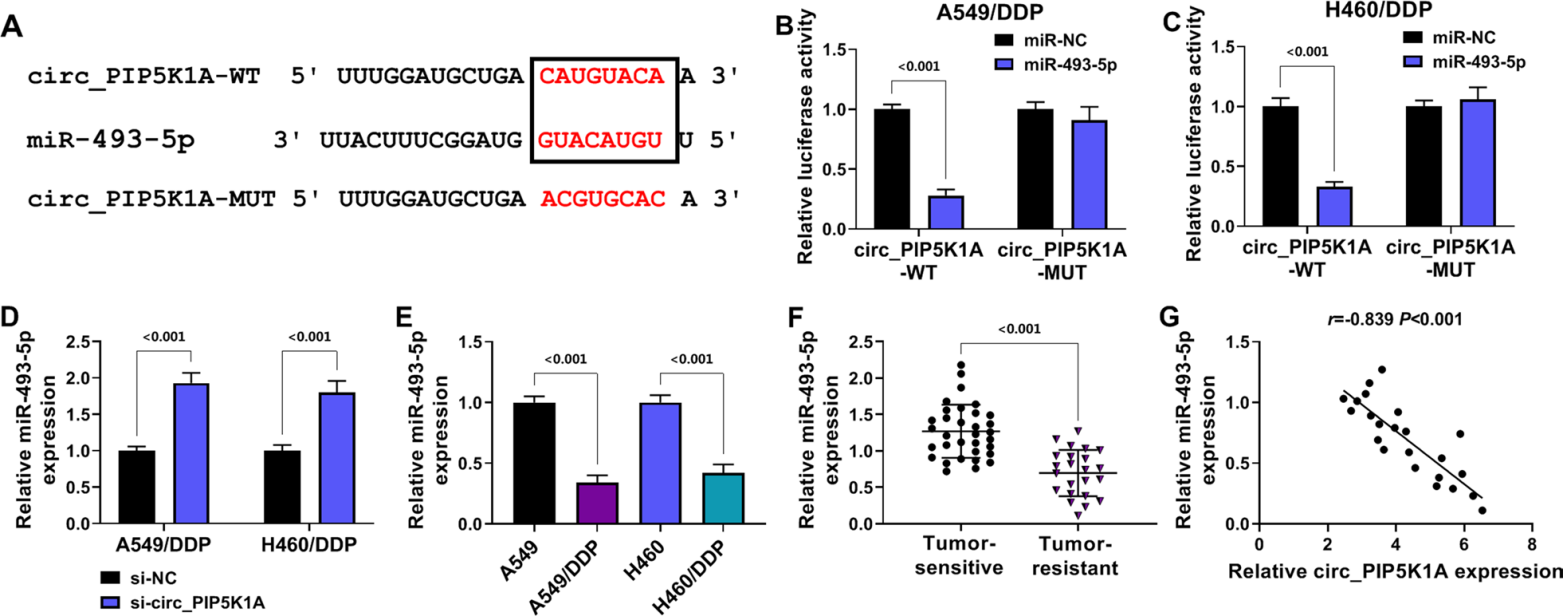
Circ_PIP5K1A interacted with miR-493-5p. **A** the binding sites between circ_PIP5K1A and miR-493-5p were exhibited by starbase. **B**, **C** dual-luciferase reporter assay was used to validate the binding between circ_PIP5K1A and miR-493-5p in A549/DDP (**B**) and H460/DDP (**C**) cells. **D** RT-qPCR was applied for the expression detection of miR-493-5p after transfection of si-NC or si-circ_PIP5K1A. **E**, **F** the level of miR-493-5p was analyzed using RT-qPCR in DDP-resistant cells (**E**) and tumor-resistant tissues (**F**). **G** Pearson’s correlation coefficient was applied for the analysis of relationship between miR-493-5p and circ_PIP5K1A in tumor-resistant tissues. ***P < 0.001

Starbase prediction exhibited the binding sites between the sequences of circ_PIP5K1A and miR-493-5p (Fig. 3A). Furthermore, the interaction relation was affirmed using dual-luciferase reporter assay. The luciferase intensity of circ_PIP5K1A-WT group was markedly inhibited following miR-493-5p transfection contrasted to miR-NC transfection (*P* < 0.001), whereas no significant difference was detected in circ_PIP5K1A-MUT group (Fig. 3B, C). RT-qPCR manifested that miR-493-5p level was increased by si-circ_PIP5K1A relative to si-NC group (*P* < 0.001) in A549/DDP and H460/DDP cells (Fig. 3D). In addition, the expression of miR-493-5p was downregulated (*P* < 0.001) in DDP-resistant NSCLC cells compared with the parental NSCLC cells (Fig. 3E) and tumor-resistant tissues relative to the tumor-sensitive tissues (Fig. 3F). Pearson’s correlation coefficient analysis indicated that the relation was negative (*r* = − 0.839, *P* < 0.001) between circ_PIP5K1A and miR-493-5p expression levels in tumor-resistant tissues (Fig. 3G). These findings suggested that circ_PIP5K1A directly combined with miR-493-5p.

### Downregulation of miR-493-5p counteracted the effects of si-circ_PIP5K1A on DDP-resistant NSCLC cells

**Fig. 4:**
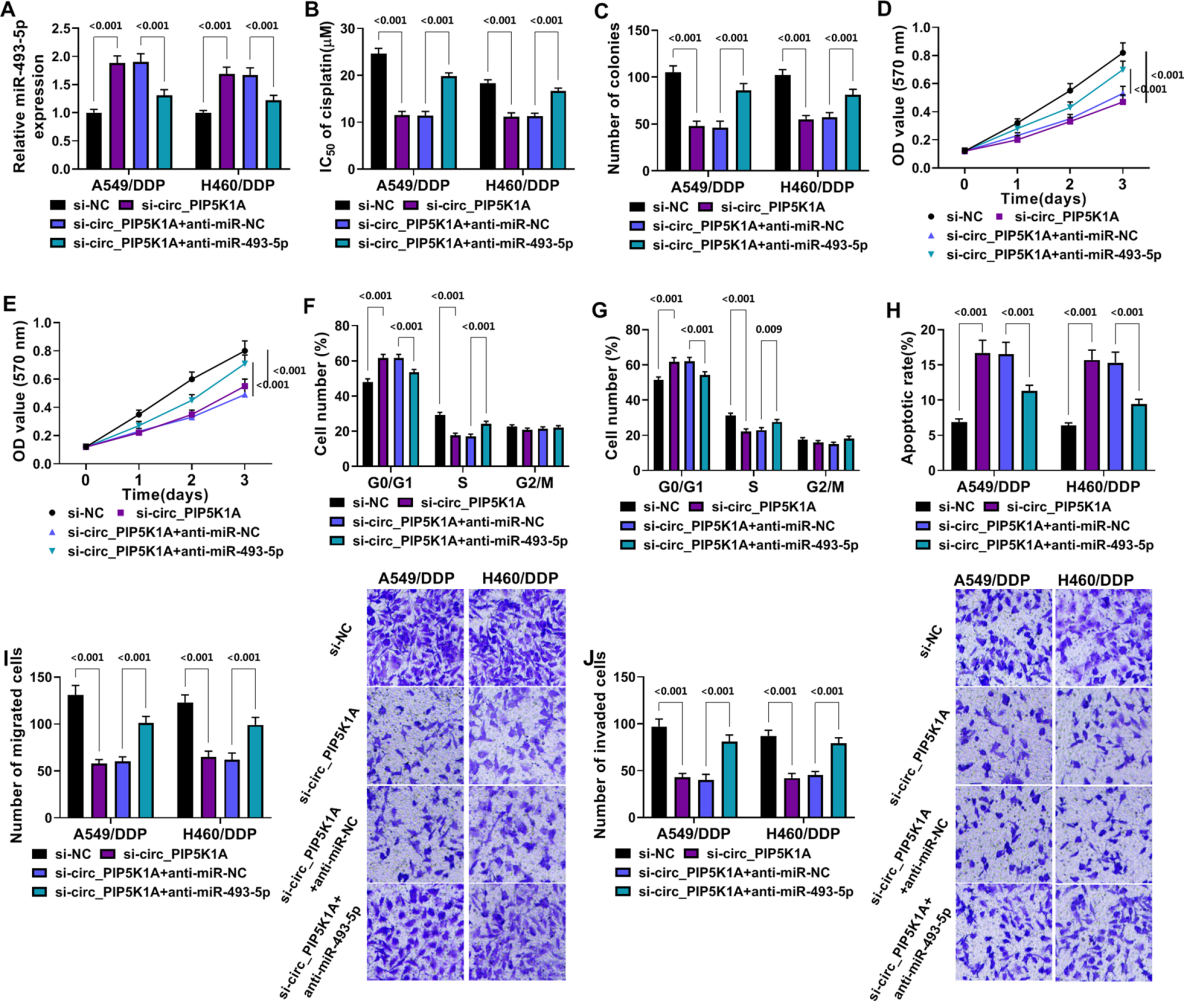
Downregulation of miR-493-5p counteracted the effects of si-circ_PIP5K1A on DDP-resistant NSCLC cells. Transfection of si-NC, si-circ_PIP5K1A, si-circ_PIP5K1A + anti-miR-NC or si-circ_PIP5K1A + anti-miR-493-5p was conducted in A549/DDP and H460/DDP cells. **A** the expression analysis of miR-493-5p was completed through RT-qPCR assay. **B** the determination of IC_50_ was performed through MTT assay. **C**–**E** the evaluation of cell proliferation and viability was carried out through colony formation assay (**C**) and MTT (**D**, **E**). **F**–**H** the examination of cell cycle (**F**, **G**) and apoptosis (**H**) was implemented through flow cytometry. **I**, **J** the assessment of cell migration (**I**) and invasion (**J**) was performed through transwell assay. **P < 0.01, ***P < 0.001

Then, we investigated the relation between circ_PIP5K1A and miR-493-5p in the malignant behaviors of DDP-resistant NSCLC cells. The expression analysis showed that anti-miR-493-5p transfection abrogated the si-circ_PIP5K1A-induced upregulation of miR-493-5p (*P* < 0.001) in A549/DDP and H460/DDP cells (Fig. 4A). The suppressive effects of si-circ_PIP5K1A on IC_50_ of DDP (Fig. 4B), cell proliferation or viability (Fig. 4C–E) and cell cycle progression (Fig. 4F, G) were countervailed after miR-493-5p level was downregulated in A549/DDP and H460/DDP cells. Meanwhile, we found that apoptosis promotion (Fig. 4H) and migration or invasion inhibition (Fig. 4I, J) caused by si-circ_PIP5K1A were also offset by miR-493-5p inhibitor (*P* < 0.001). Altogether, the function of circ_PIP5K1A was achieved by the negative regulation of miR-493-5p in DDP-resistant NSCLC cells.

### Circ_PIP5K1A knockdown downregulated the ROCK1 expression by releasing miR-493-5p

**Fig. 5:**
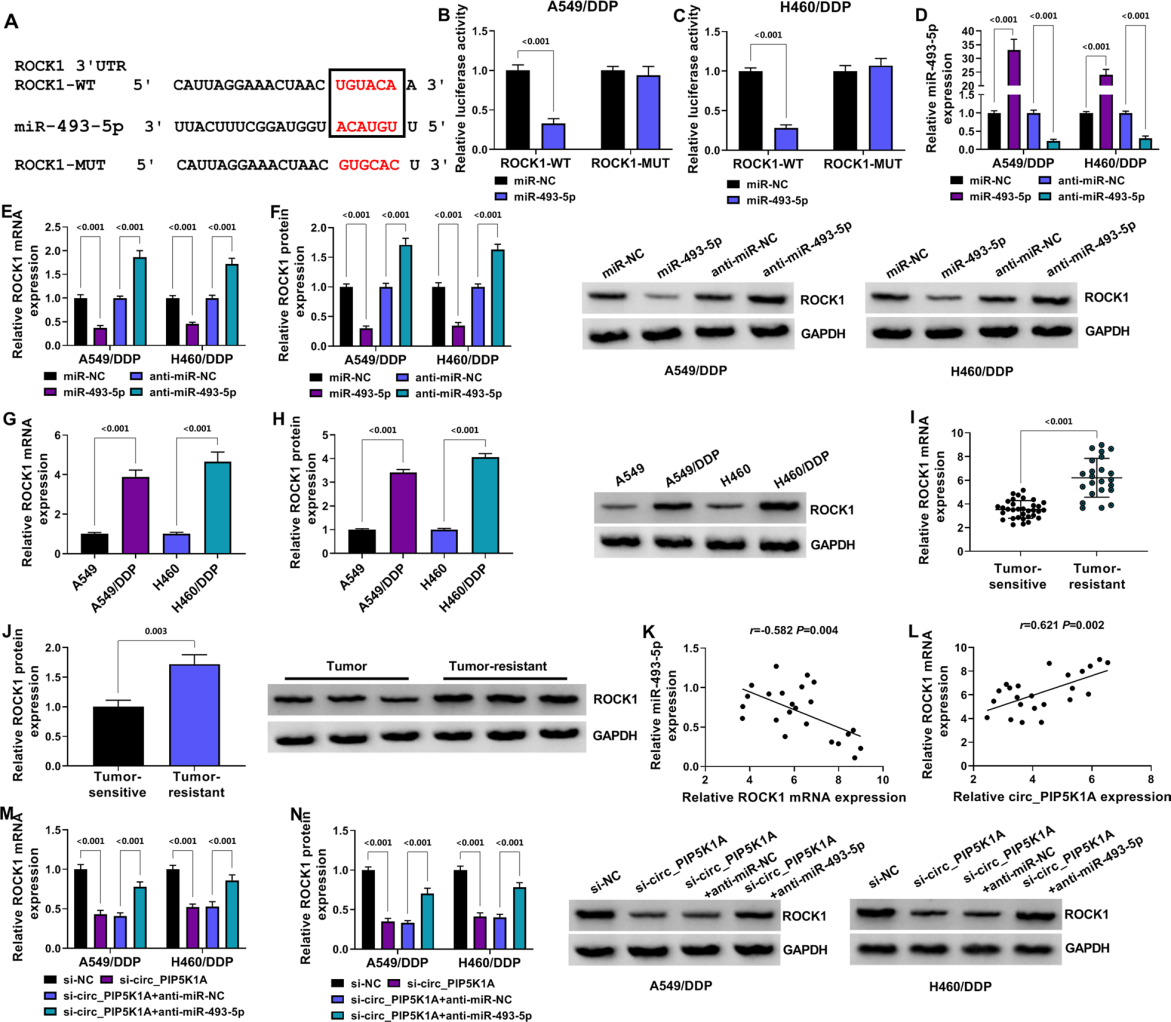
Circ_PIP5K1A knockdown downregulated the ROCK1 expression by releasing miR-493-5p. **A** the online starbase indicate the binding sites between miR-493-5p and ROCK1 3′UTR. **B**, **C** the combination between miR-493-5p and ROCK1 3′UTR was analyzed through dual-luciferase reporter assay. **D** the transfection efficiencies of miR-493-5p and anti-miR-493-5p were assessed using RT-qPCR. **E**, **F** the effects of miR-493-5p overexpression or inhibition on ROCK1 mRNA (**E**) and protein (**F**) levels were detected via RT-qPCR and Western blot. **G**–**J** RT-qPCR and Western blot were performed for ROCK1 detection in DDP-resistant cells (**G**, **H**) and tumor-resistant tissues (**I**, **J**). **K**, **L** the correlations between ROCK1 and miR-493-5p (**K**) or circ_PIP5K1A (**L**) in tumor-resistant tissues were analyzed using Pearson’s correlation coefficient. **M**, **N** ROCK1 mRNA (**M**) and protein (**N**) levels were assayed by RT-qPCR and Western blot in A549/DDP and H460/DDP cells transfected with si-NC, si-circ_PIP5K1A, si-circ_PIP5K1A + anti-miR-NC or si-circ_PIP5K1A + anti-miR-493-5p. **P < 0.01, ***P < 0.001

Starbase also predicted that the 3′UTR of ROCK1 sequence contained the miR-493-5p binding sites (Fig. 5A). Overexpression of miR-493-5p resulted in the luciferase signal inhibition of ROCK1-WT group instead of ROCK1-MUT group (*P* < 0.001) in A549/DDP and H460/DDP cells (Fig. 5B, C). RT-qPCR affirmed that the overexpression and inhibitory efficiencies of miR-493-5p and anti-miR-493-5p were prominent compared with miR-NC and anti-miR-NC groups (*P* < 0.001) (Fig. 5D). ROCK1 mRNA and protein levels were decreased in miR-493-5p group relative to miR-NC group (*P* < 0.001), while the opposite expression change of ROCK1 was induced by anti-miR-493-5p relative to anti-miR-NC group (*P* < 0.001) (Fig. 5E, F). Thus, miR-493-5p could negatively regulate the level of ROCK1. In comparison to the parental cells and tumor-sensitive tissues, ROCK1 was upregulated (*P* < 0.001) in DDP-resistant cells (Fig. 5G, H) and tumor-resistant tissues (Fig. 5I, J). There was a negative relation (*r* = − 0.582, *P* = 0.004) between miR-493-5p and ROCK1 (Fig. 5K) but a positive correlation (*r* = 0.621, *P* = 0.002) between circ_PIP5K1A and ROCK1 (Fig. 5L) in tumor-resistant samples. RT-PCR and Western blot manifested that ROCK1 expression was reduced by si-circ_PIP5K1A compared to si-NC group (*P* < 0.001), while ROCK1 was upregulated in si-circ_PIP5K1A + anti-miR-493-5p group contrasted with si-circ_PIP5K1A + anti-miR-NC group (*P* < 0.001) (Fig. 5M, N). These results demonstrated that circ_PIP5K1A triggered the upregulation of ROCK1 expression via targeting miR-493-5p.

### MiR-493-5p acted as a sensitizer of DDP and tumor inhibitor in DDP-resistant NSCLC cells by inducing ROCK1 downregulation

**Fig. 6:**
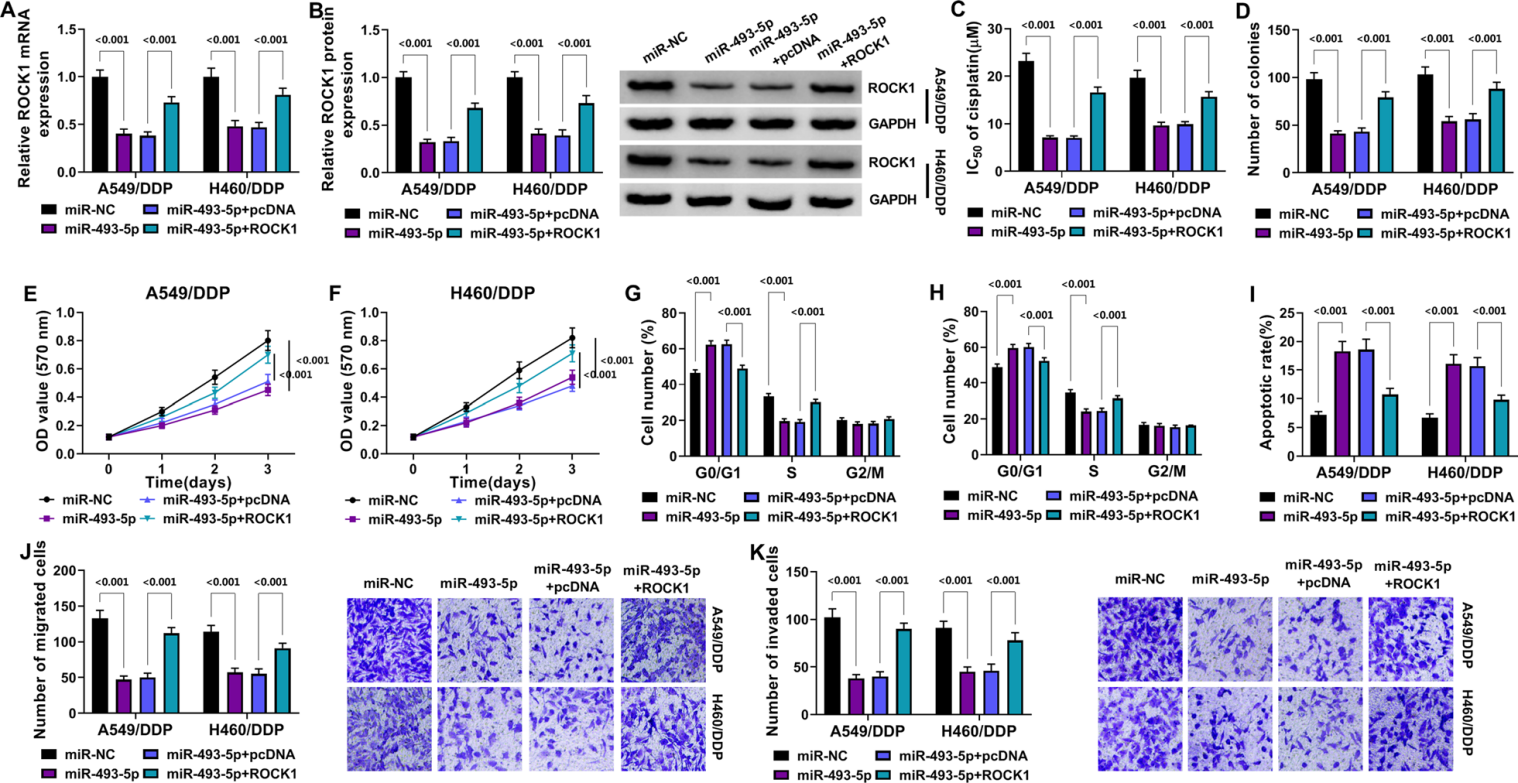
MiR-493-5p acted as a sensitizer of DDP and tumor inhibitor in DDP-resistant NSCLC cells by inducing ROCK1 downregulation. Transfection of miR-NC, miR-493-5p, miR-493-5p + pcDNA or miR-493-5p + ROCK1 was carried out in A549/DDP and H460/DDP cells. **A**, **B** ROCK1 expression was quantified using RT-qPCR and Western blot. **C** IC_50_ of DDP was measured via MTT assay. **D**–**F** cell proliferation and viability were detected via colony formation assay (**D**) and MTT (**E**–**F**). **G**–**I** cell cycle (**G**, **H**) and apoptosis (**I**) were analyzed by flow cytometry. **J**, **K** cell migration (**J**) and invasion (**K**) were evaluated using transwell assay. ***P < 0.001

The role and mechanism of miR-493-5p were explored in DDP-resistant cells. The miR-493-5p transfection induced the ROCK1 mRNA and protein expression reduction by comparison with miR-NC transfection (*P* < 0.001), then this expression change was relieved by transfection of ROCK1 relative to pcDNA transfection (*P* < 0.001) (Fig. 6A, B). IC_50_ of DDP (Fig. 6C), cell proliferation and viability (Fig. 6D–F) in A549/DDP and H460/DDP cells were inhibited by miR-493-5p compared with miR-NC group (*P* < 0.001), which was notably counterbalanced by ROCK1 but not pcDNA (*P* < 0.001). The introduction of ROCK1 also attenuated the miR-493-5p-mediated cell cycle retardation (Fig. 6G, H), cell apoptosis enhancement (Fig. 6I) and migration or invasion suppression (Fig. 6J, K) contrasted to the introduction of pcDNA (*P* < 0.001). All in all, the inhibitory effects of miR-493-5p on DDP resistance and NSCLC development were associated with the downregulation of ROCK1.

### Circ_PIP5K1A regulated DDP sensitivity to NSCLC in vivo by the expression regulation of miR-493-5p and ROCK1

**Fig. 7:**
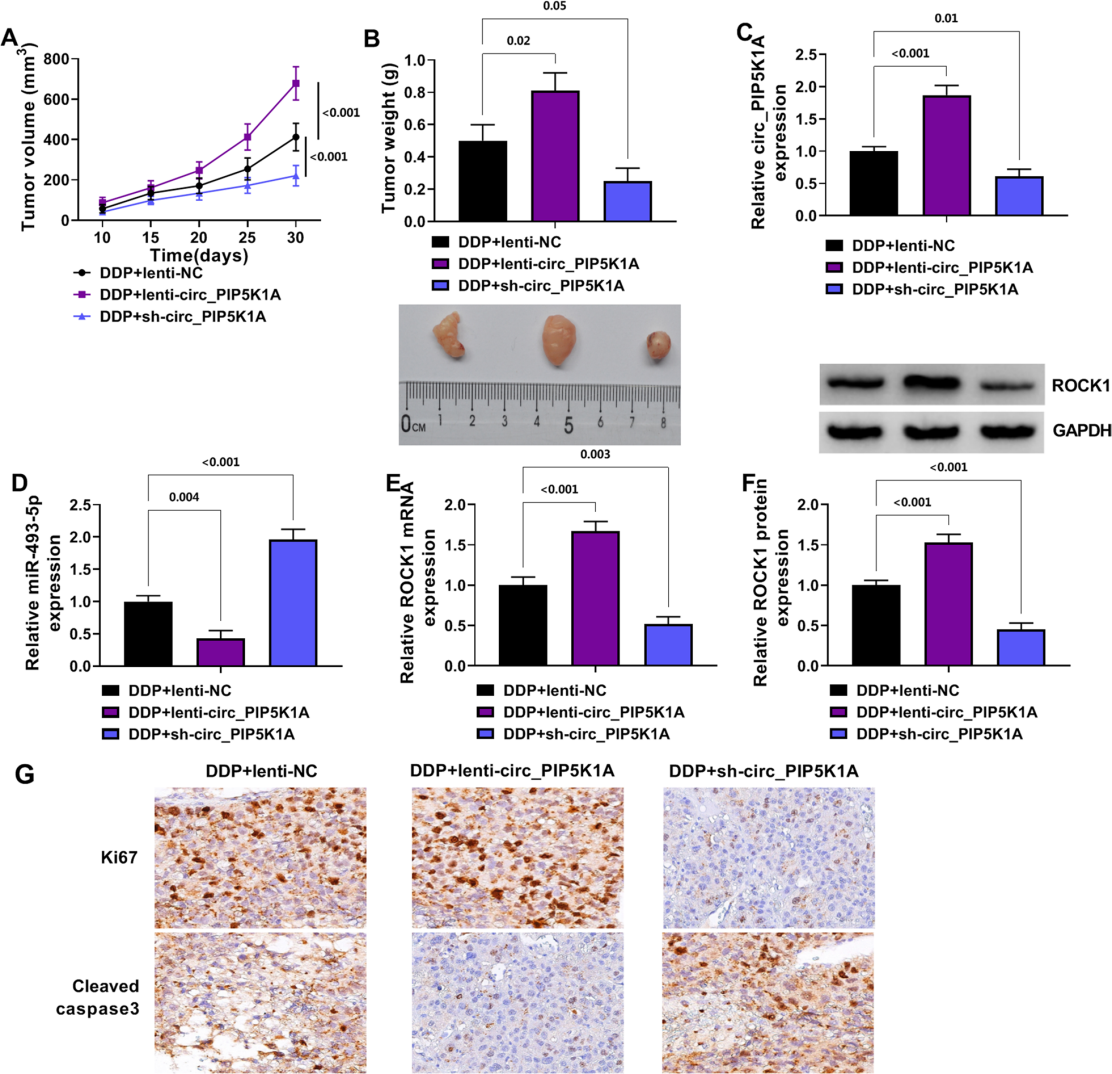
Circ_PIP5K1A regulated DDP sensitivity to NSCLC in vivo by the expression regulation of miR-493-5p and ROCK1. **A**, **B** tumor volume (**A**) and weight (**B**) were determined in DDP + lenti-NC, DDP + lenti-circ-PIP5K1A and DDP + sh-circ_PIP5K1A groups. **C**, **D** the levels of circ_PIP5K1A and miR-493-5p in tumor tissues were examined by RT-qPCR. **E**, **F** ROCK1 mRNA and protein detection was performed via RT-qPCR and Western blot in tumor tissues. **G** Ki67 and Cleaved caspase3 levels in tumor tissues were analyzed using IHC assay. *P < 0.05, **P < 0.01, ***P < 0.001

Xenograft model was established after mice were injected with transfected A549/DDP cells and DDP. Tumor volume was promoted in DDP + lenti-circ_PIP5K1A group (*P* < 0.001) but inhibited in DDP-sh-circ_PIP5K1A group (*P* < 0.001), relative to DDP + lenti-NC group (Fig. 7A). Also, tumor weight was increased in DDP + lenti-circ_PIP5K1A group (*P* = 0.02) while reduced in DDP-sh-circ_PIP5K1A group (*P* = 0.05), compared to DDP + lenti-NC group (Fig. 7B). Circ_PIP5K1A level was higher in DDP + lenti-circ_PIP5K1A group than that in DDP + lenti-NC group (*P* < 0.001), but circ_PIP5K1A downregulation was evoked by treatment of DDP + sh-circ_PIP5K1A contrasted with treatment of DDP + lenti-NC (*P* = 0.01) (Fig. 7C). In addition, DDP + lenti-circ_PIP5K1A led to the inhibitory effect on miR-493-5p expression (*P* = 0.004) but DDP + sh-circ_PIP5K1A elevated the miR-493-5p level (*P* < 0.001) relative to DDP + lenti-NC in tumor tissues (Fig. 7D). The mRNA and protein levels of ROCK1 were upregulated in DDP + lenti-circ_PIP5K1A treatment group compared with DDP + lenti-NC treatment group (*P* < 0.001), whereas the downregulation of ROCK1 was detected in mRNA (*P* = 0.003) and protein (*P* < 0.001) expression in DDP + sh-circ_PIP5K1A treatment group (Fig. 7E, F). The results of IHC assay revealed that Ki67 protein level was upregulated by circ_PIP5K1A overexpression but sh-circ_PIP5K1A evoked the protein reduction of Ki67, and circ_PIP5K1A inhibited the protein expression of Cleaved caspase3 (Fig. 7G). Taken together, circ_PIP5K1A increased the sensitivity of tumor to DDP in vivo through modulating the miR-493-5p and ROCK1 expression.

## Discussion

The therapeutic outcomes of cancer patients have been largely affected by chemoresistance. Herein, our results in vitro and in vivo manifested that knockdown of circ_PIP5K1A could enhance sensitivity of NSCLC to DDP. Circ_PIP5K1A might be applied as a biomarker to improve the DDP therapy for NSCLC patients. The molecular mechanism of circ_PIP5K1A in the regulation of resistance was also disclosed for the first time.

The covalent closed structures confer circRNAs the high stability in human eukaryotes [19]. Our data showed that circ_PIP5K1A was more stable than linear PIP5K1A after treatment with RNase R and Actinomycin D in both A549/DDP and H460/DDP cells. CircRNAs have pivotal roles in various kinds of biological processes, including carcinogenesis, cancer progression and drug resistance [20]. Qian et al. discovered that circ-G004213 significantly elevated the DDP sensitivity in liver cancer via regulating the miR-513b-5p/pre-mRNA processing factor 39 (PRPF39) levels [21]. Wei et al. reported that circSAMD4A increased doxorubicin resistance in osteosarcoma cells through the effect on miR-218-5p/krüppel-like factor 8 (KLF8) axis [22]. Circ_CELSR1 overexpression also promoted the chemoresistance of ovarian cancer cells to paclitaxel via mediating the salt inducible kinase 2 (SIK2) level by targeting miR-149-5p [23]. The expression analysis revealed that circ_PIP5K1A was significantly upregulated in tumor-resistant tissue samples and DDP-resistant NSCLC cells, which implied that circ_PIP5K1A might be involved in the resistance of DDP in NSCLC. As expected, we found that circ_PIP5K1A downregulation repressed the IC_50_ of DDP in resistant NSCLC cells. Cellular behavior analysis has shown that silence of circ_PIP5K1A induced the inhibitory effects on cell proliferation, cell viability, cell cycle progression and cell migration/invasion but the stimulative effect on apoptosis in DDP-resistant cells. All these findings suggested that circ_PIP5K1A inhibition reduced DDP resistance to further impede the progression of NSCLC.

Many studies have validated that the regulatory functions of circRNAs were associated with miRNA/mRNA signal networks. For instance, circFBXW7 played a tumor-inhibitory role in lung adenocarcinoma via sponging miR-942-5p and increasing the BARX homeobox 2 (BARX2) expression [24]. CircUBE2D2 enhanced cell proliferation and doxorubicin resistance in triple-negative breast cancer cells by controlling miR-512-3p/cell division cycle associated protein-3 (CDCA3) axis [25]. CircRNA_001275 increased the wingless-type protein 7a (Wnt7a) expression by competitively sponging miR-370-3p to facilitate the resistance of DDP to esophageal cancer cells [26]. In this study, circ_PIP5K1A upregulated the ROCK1 expression through sponging miR-493-5p. The regulation of circ_PIP5K1A knockdown was ascribed to the miR-493-5p level upregulation. Also, miR-493-5p inhibited chemoresistance and oncogenesis of DDP-resistant cells by targeting ROCK1. Animal assay further revealed that circ_PIP5K1A expression reduction contributed to DDP sensitivity by affecting the miR-493-5p/ROCK1 axis in vivo.

There are still some limitations in the current study. For example, the further experiment of circ_PIP5K1A/miR-493-5p/ROCK1 axis in regulating DDP resistance in vivo needs to be performed. Additionally, whether circ_PIP5K1A could regulate the signaling pathways via the miR-493-5p/ROCK1 axis remains unknown. ROCK1 has inactivated the LATS2-JNK pathway and PTEN/PI3K/FAK pathway in NSCLC progression regulation [27, 28]. Exploring the signaling pathways in the downstream of circ_PIP5K1A/miR-493-5p/ROCK1 will be beneficial for the better understanding of the functional mechanism behind circ_PIP5K1A.

## Conclusion

In conclusion, circ_PIP5K1A/miR-493-5p/ROCK1 axis was implicated in the regulation of DDP resistance and malignant behaviors in NSCLC cells and murine model. This study might show a novel perspective for increasing DDP sensitivity, with circ_PIP5K1A as a potential biological marker.

## Data Availability

Please contact the correspondence author for the data request.
